# The Platonic Dialogues
†*The Platonic Dialogues for English Readers*. By William Whewell, D.D. Vol. i. Cambridge. 1859.


**Published:** 1860-04-01

**Authors:** 


### Art. II.—THE PLATONIC DIALOGUES.†

"Every one," writes Dr. Whewell, "that has any tinge of literature has heard of Socrates and Plato, who lived at Athens at the time of its greatest glory, when philosophy had its birth there." Yet it may be safely said, that until very lately few either

† *The Platonic Dialogues for English Readers.* By William Whewell, D.D. Vol. i. Cambridge. 1859.

knew or had the opportunity of knowing these men as they could alone be rightly known, by the writings of the latter one and of the contemporaries of both. Not many of us, indeed, have that familiarity with ancient Greek which would enable us to overmaster with comfort the difficulties which beset the original text of Plato's writings, the principal source from which we must draw our knowledge of the father of philosophy and of his chiefest scion. Not many, indeed, it may perhaps be asserted, have had the courage to seek, or even if they had sought, would have found that familiar knowledge of the men as MEN, which the ordinary, thoughtful reader might above all things desire, in the recondite dissertations which abound upon their characters and philosophy. Socrates and Plato the PHILOSOPHERS were to be seen depicted everywhere; but Socrates the gossip and Plato the gentleman, Socrates the inelegant and Plato the dandy, Socrates the blunt and Plato the refined, Socrates the Marsyas and Plato the Apollo-begotten—who could know them, except by seeking for himself those slighter traits of character which crop out plentifully in the recorded conversations of the one and the writings of the other?

When Mr. Bohn a little while ago published, at a very moderate cost, translations of the whole of Plato's works, he gave every English reader an opportunity of acquiring this knowledge; and had he done no other service to the general literature of the country, he would have richly deserved our gratitude for this one. And it is certain that the boon was appreciated in a great degree. The volumes may be seen well thumbed in many an operative's library, and we have heard of coalminers in the north, sitting round the blazing fire after the day's work was done and hearkening with wrapt attention to the wondrous language of the dæmon-like philosopher. Nay, we have listened to weavers and mechanics in the pulpit and on the platform urge their arguments home with his teachings. And who, who has once commenced to read his dialogues has not quickly ceded to their weird-like influence? You forget the writer; you forget the time of writing. It is a thing of to-day, not of two thousand years ago, and of the older Athens. What though, every now and then, you stumble in the obscurity of the dawn of pure thought, or trip over an ornate paganism, it is but for a moment. You are reading a philosophical fairy tale, and the curious garnishments of mythology, and the, at times, child-like simplicity of illustration, all serve but as a sort of piquant framework in which is set a moral so pure, so deep, that at every moment it touches and awakens into activity your better feelings. This is the feeling of the mere reader for amusement, and this derived from the reading of Mr. Bohn's edition, notwithstanding that in it we have often to contend with the harshness of a literal rendering. Nor, indeed, need we wonder at such a result, for it is with the Platonic dialogues now as it was with them when they appealed to the Athenians of old, and to the mid-time between Plato and ourselves. It is but one of many illustrations that man at least is the same at the time being as he ever was, and that we have not as yet any new version of the "the grand old legend of humanity." Olympiodorus writing twelve hundred years ago, tells us that when Plato was about to die, he having made many admirers and benefited the most of them, dreamed that he had become a swan, and that flying from tree to tree, he caused the greatest trouble to the bird-limers. Simmias, the Socratic philosopher, expounded the dream after this fashion, to wit, "that Plato would not be caught by those, who coming after him wished to interpret him. For the interpreters who desired to lay hold of the meaning of the ancients were like bird-limers. And truly Plato has not been caught, since we may take his words like those of Homer, in a physical, moral, ethical, or theological sense, to speak simply, in a variety of senses. For the souls of the poet and the philosopher were said to be altogether in harmony, and hence one may take the words of both in various senses." This is the secret of the whole matter. Plato's words, impressed with a mighty and mysterious genius, LIVE, they do indeed constitute a language which, as Carlyle would say, is the "Flesh-Garment, the Body of Thought;" and he who reads them listens as it were to the newly-told tale of freshly-enacted events, while the thoughts the words convey will most surely rouse up within him, be he learned or unlearned, wise or foolish, philosopher or poet, theologist, moralist, or the mere man of the world, somewhat or other of those better feelings which give nobility to the soul. Most aptly did Simmias, as Olympiodorus records, class Homer and Plato as kindred souls. A modern poet has written that—

"The poet's pen is the true divining rod
Which trembles towards the inner founts of the feeling;
Bringing to light and use, else hid from all,
The many sweet, clear sources that we have
Of good and beauty in our own deep bosom,
And marks the variations of all mind
As does the needle an air-investing storm." *

And so with Plato's pen. It wrote of themes that touch the very spring-head of our feelings, and in such wise, that they respond as readily at the present time as they would have done had we listened to the philosopher himself, if not, indeed, more so. For standing in the noon-day sun of Christianity, we see clearly and fully now those great truths which both Socrates

* Bailey's *Festus*.

and Plato could only look at through a haze of mythology. Such noble doctrines as they taught come fully home to us, but with them to what extent was their aim diverted by the distorting and warping medium of polytheism?

Many general readers have, however, doubtless been debarred from reading the Platonic Dialogues on account of their prolixity. Six closely-printed octavo volumes of philosophical writings—such being the bulk of Mr. Bohn's edition of Plato's works—it must be confessed require pretty tolerable reading capacity on the part of a general reader to grapple with, notwithstanding the inducements which may be held out to him by those who, having achieved the work, rejoice in the new world of thought opened out to them. But let the faint-hearted now also sing *Io Pæan*, since, thanks to Dr. Whewell, a series of the Platonic Dialogues—a first instalment only, we sincerely hope—is from henceforth accessible to them, decked in sterling and euphonious English, and shorn of all prolixity and obscurity, by those portions of the dialogues which are least essential to the arguments being given in abstract. Moreover, each dialogue is accompanied by sundry valuable comments on the nature and character of the reasonings it contains, on the time at which it was probably written, and on its genuineness. We are taught also all things necessary to understand who were the individuals represented as interlocutors in the dialogues, while the character of the chief one, Socrates, and his teachings, are further elucidated by collateral testimony quoted from writers contemporary with Plato. In truth, the book is a dainty morsel for the scholar, and an admirable manual of the Platonic writings for the general reader, and we err greatly in our estimate of its attractiveness, if it does not become a very popular work. Let us justify this opinion in some degree, assuring our readers that if we fail in so doing, the fault is with us, and not with Dr. Whewell's translation.

He, in the present volume, confines himself to what he has termed the *Dialogues of the Socratic School*. It may be well to give his reason for this classification. He writes:—

"The subject of philosophy includes a vast multiplicity of trains of thought, of the most different kinds, reaching from the first questions asked by an intelligent and inquisitive child, to subtle inquiries which task the intellects of the wisest man, and which often bewilder the clearest heads. The Platonic Dialogues present to us specimens of these different kinds of inquiries; and in order to understand the dialogues we must, in presenting them to the English reader, mark them as belonging to one or another of these classes, according as they really do so. Where the discussion runs into subtleties which are now of no philosophical interest, we may abridge or omit them, in order that the general reader may not be repelled from that which has really a general interest. On the other hand, where the conversation is really concerning difficulties which belong to the infancy of systematic thinking,—concerning ambiguities of words and confusions of notions which may perplex children but which any thoughtful man can see through,—we must take care not to mislead our readers by speaking as if these juvenile exercises of thought had some profound and philosophical meaning. We shall find that this caution is by no means unneeded.

"Since the Platonic Dialogues are of such various kinds, they may on this ground be separated into different classes; as they may also on other grounds, for instance, their relation to the fate of Socrates the main character of their drama; or their connexion with the progress of opinion in the mind of Plato their author. But the present volume will contain a single class of them, which may on all these grounds be regarded as the earliest, and which we shall call *Dialogues of the Socratic School.*

"In this designation one main fact implied is that Socrates in his conversation had some prevailing and habitual ways of thinking and talking, which are prominent in some of the Platonic Dialogues, while in others the train of thought and speculation appears to belong rather to Plato himself than to Socrates."

Socrates, indeed, holds so eminent a position in these dialogues, and he is so vividly depicted, that do as we may, we are apt at every turn to forget the writer in the thing written. Socrates speaks to us rather than Plato; and it is hard to disabuse ourselves of the notion that the dialogues are the literal records of actual occurrences, and not imaginary ones. It will be prudent, therefore, before we string together a few illustrations of the character and teachings of the Platonic Socrates to say somewhat of Plato himself.

We have spoken of him as *dæmon-like.* Let not the phrase be misapprehended. We mean dæmon in the Platonic sense of the word, that is, neither wholly God nor wholly man, but partaking of the nature of both, and not the dæmon of the Middle Ages, the plebeian of the infernal regions, for Wierius tells us that dæmons are "les roturiers de l'enfer."* That Plato was looked upon as dæmon-like in his own age, is evident from the fabulous account which is given of his conception, for it is recorded that he was begotten by Apollo upon the, until that time, virgin Perictione. We also learn from a delicious little fable, that while the philosopher was yet in babyhood, his mother and putative father Aristo wended their way to Mount Hymettus to sacrifice there to Apollo, Pan, and the Nymphs, and while the rites were being duly performed, the god-begotten infant was placed on the ground upon the sweet-smelling herbs. Then, while the little thing tossed its limbs in its father's beams, the bees which made the air

* Colin de Plancy: *Dictionnaire Infernal.*

tremulous with their busy hum, flocked to the baby's mouth and deposited upon its lips their golden honey, thus foreshowing the future irresistible charm which would flow from its eloquent tongue. At first named Aristocles, he was subsequently styled *Plato,*—he of the broad shoulders and noble brow, or, as others will have the word to signify, he of the bold, open, and flowing style. He was trained in all the accomplishments of an Athenian youth, and had diligently studied the arts of painting and poetry. He composed an epic poem, but he cast it into the flames when he had compared it with Homer; he wrote also a dramatic piece, but the day before it was exhibited at the theatre, he happened to hear Socrates conversing, and was so charmed with his discourse, that from that time forth he gave himself up solely to the study of philosophy. He was in his twentieth year when he became a pupil of the father of philosophy, and his readiness of apprehension was quickly manifested by his grafting from time to time additional thoughts upon those of Socratic teaching, somewhat to the annoyance of Socrates and his pupils, as it is said. From Socrates he first learned the power and value of his previous training, and how rightly to *think,* and with such results that the fame of Plato's instructions is as conspicuous now as when he taught at Athens with so great skill that young and old, the noble and the humble, nay even women in male attire, crowded to listen to him. And yet the charm of his teaching depended upon his language and thoughts alone, for it is said that his voice wanted both strength and tone. He was a man of graceful and manly bearing, grave, modest, and quiet in his demeanour. He was never seen to laugh excessively. His gravity of aspect was a subject of jest among the comic writers of the time. One wrote :—

"Oh Plato! how thou nothing know'st except
To wear a scowling look, and eyebrows raise,
Like one who's bilious, with a solemn air."*

He shunned as well the bluntness of manner as the carelessness in dress which marked Socrates, and he has been accused of being extravagant in attire and of inducing his pupils also to be so, although the author of the charge asserts also that he was a sycophant for money. Ephippus the comic poet writes :—

"Then some ingenious young man rising up,
Some pupil of the New Academy,
Brought up at Plato's feet and those of Bryso,
That bold, contentious, covetous philosopher,—
And urged by strong necessity, and able,
By means of his small-wages-seeking art

* Amphis in *Dixidemides,* Diogenes Laertius.—Burges.

To speak before th' assembly, in a manner
Not altogether bad; having his hair
Carefully trimmed with a new-sharpened razor,
And letting down his beard in graceful fall,
Putting his well-shod foot in his neat slipper,
Binding his ankles in the equal folds
Of his well-fitting hose, and well protected
Across the chest with the breastplate of his cloak,
And leaning in a posture dignified,
Upon his staff, said, as it seems to me,
With mouthing emphasis, the following speech,
More like a stranger than a citizen,
'Men of the land of wise Athenians.'"

Athenæus, who quotes this fragment in the Deipnosophists,* also sums up many little things to show that Plato did not lack a spice of ill-nature towards his contemporaries in learning; and further asserts that "altogether he displayed the feelings of a stepmother towards all the pupils of Socrates." There is not wanting ground for this belief, and it is also not improbable that the philosopher was tinged somewhat with the gross licentiousness of the period in which he lived. These are the spots on the sun. He never married, and he lived until he had attained his eighty-first year. His death occurred, according to some writers, while he was writing; according to others, while he was at a marriage-feast. The Athenians buried him with all honour, and upon his tomb was inscribed the couplet:—

"These two, Æsculapius and Plato, did Apollo beget,

One that he might save the soul, the other the body."

There is a story, that when Socrates was about to receive Plato, the former dreamed that a swan without wings settled upon his knees, and becoming fledged on the instant, flew up to the sky, singing so sweetly that all who heard it were enchanted. The next day, Plato came to Socrates, who thereupon exclaimed, "This is the bird." Let us learn somewhat of the man who fixed to Plato his wings, and of the mode in which this was effected. These are old stories, but stories of which the world never tires.

Dr. Whewell describes Socrates as "a private Athenian citizen, who, like other citizens, had served in various public offices; served too as a soldier, and served well; and whose favourite and constant employment it was to spend his time in the streets, in the market-place, in the open shops, wherever the Athenians lounged or gossiped. There he got hold of one person after another, and questioned and cross-questioned him, and argued with him in the most pertinacious and unsparing manner. His appearance gave point to his copious and eager speech. His counte-

* Yonge's translation.

nance was plain, amounting to grotesque, but vigorous, vivacious, and good-humoured in a striking degree; his nose was flat, his mouth wide, his lips large, his forehead broad, with strong arches of wrinkles over each eyebrow, giving him a look of humorous earnestness; his figure solid but ungraceful, and his dress of the plainest materials." *

With this vantage-ground, turn we now to the Socrates of Plato.

Socrates, Plato tells us,† was most like those sitting figures of Silenus, having reeds or flutes in their hands, which one might see in the workshops of the statuaries, ungainly without, but when opened down the middle, they were found to contain within them statues of gods. Again, he is said to be like unto the Satyr Marsyas, but was a piper more wonderful than he, for he charmed men "through instruments by a power proceeding from the mouth," causing those who heard him to be spell-bound by the music, but Socrates effected the very same thing by naked words without instruments. "We therefore," Alcibiades is represented as saying, "when we hear another person, although a good speaker himself, pronouncing the speeches of others, not a single hearer, so to say, pays any regard to them; but when any one hears you (Socrates), or your discourses spoken by another, although he is a wretched speaker, yet, whether a woman, or a man, or a lad is the auditor, we are astonished and spell-bound. I, therefore, gentlemen, unless I seemed to be very much in liquor, would tell you upon oath, what I have suffered by the discourses of this man, and am suffering even now. For when I hear him, my heart leaps much more than that of the Corybantes; and my tears flow forth through his discourses. I see, too, many others suffering in the very same way. But when I hear Socrates, and other excellent orators, I think, indeed, that they speak well, but I suffer nothing of this kind; nor is my soul agitated with tumult, nor is it indignant, as if I were in a servile state. But by this Marsyas here, I am often so affected that it appears to me I ought not to live, while I am in such a state."

So also do we find equally forcible expressions respecting the charm of the philosopher's conversation and teaching in the *Meno*, the dialogue on Virtue. Meno says:—

"Ah, Socrates, before I was in your company I had heard of your way, that you do nothing but doubt yourself, and make others doubt. And accordingly, I now find that you are absolutely a magician who cast your charms and enchantments over me, so that I am filled with doubts. And in truth, if I may be allowed such a joke, you seem to me to resemble, both in your looks and in your ways, that flat-fish the

* Page 6. The subsequent references, if not otherwise stated, will be to Dr. Whewell's work.

† *The Banquet*, Burges's transl.

numbing-ray. That creature benumbs the limbs of any one who approaches and touches it: and you seem to have produced a like effect upon me; you have benumbed me. I am benumbed, body and soul, and do not know how to answer you. And yet I have heretofore ten thousand times made many speeches about Virtue to many persons, and right well too, as I then thought. I think you do well to stay at home, and not to travel into foreign lands. If you were to go into another city, and do what you do here, you would soon be packed off as a wizard."

Mark now the pleasantry of the rejoinder:—

"Soc. You are a rogue, Meno. You had nearly taken me in.—How so, Socrates?—Soc. I know why you make a comparison of me.—Men. And why, do you think?

"Soc. That I might in return make a comparison of you. I know the way of all handsome people, they are fond of being told what they are like: they have their advantage in it; for the likenesses of the Beautiful are beautiful. But I will not retaliate by making a comparison of you. But as to the numbing-ray, if it benumbs others by being itself benumbed, I *am* like it: but if this is not the case, I am not. For it is not that seeing my own way clearly I puzzle other persons; but entirely otherwise, that being puzzled myself, I make other persons puzzled too."

"Hear too from me on other points, how like he (Socrates) is to what I have compared him," remarks Alcibiades, "and what a wonderful power he possesses." We are told that he was heedless of and knew nothing concerning his own figure. "Is not this Silenus-like? For he is invested with this externally like a carved Silenus; but when he is opened inwardly, with how great temperance, think you, fellow-tipplers, is he filled?" He heeded not beauty lasciviously; he despised wealth, neglecting money-making, the care of his household, public offices, and private engagements, "thinking himself too honest a man to escape ruin if he engaged in such."* He passed, it is said, his whole time indulging irony and jests against mankind, and Plato represents him as saying in the *Apology*:—

"'Why is it that some are pleased to spend much time in my company? You have heard already, men of Athens. I have told you the whole truth of the matter. Men are pleased to hear those exposed who think that they are wise, and are not so; for it is an exhibition not unamusing. And to do this, is my task imposed by the God, by oracles and dreams, and in all ways, like any destiny of any other man by which he has his appointed work.'"†

Again, in the same dialogue:—

"'Perhaps it may appear absurd that I go about giving advice to particular persons and meddling with everybody, and yet that I do not come forwards before your public assemblies and give my advice

* *Apology*, p. 323. † *Ib.* 318.

about matters of state. The cause of this is, that which I have often said and you have often heard, that I have a Divine Monitor of which Meletus in his indictment makes a charge in so extravagant a manner. This Monitor I have had from my boyhood—a voice which warns me, which restrains me constantly from what I am about to do, but never urges me on to do. This was what stood in the way of my undertaking public affairs. When you may be well assured that if I had engaged in public business I should long ago have perished, and should have done no good either to you or to myself. And be not offended with me when I tell you the truth. No man can long be safe who, either to you or to any other democratic body, opposes himself frankly, and resists wrong and illegal things being done by the city. It is necessary that he who really fights for what is right, if he is to be safe even for a short time, should be in a private, not in a public station."

In the *Meno*, Anytus speaks thus :—

"'Socrates, you seem to me to be prone to speak ill of men very lightly. If you will take my advice, I would recommend you to take care of what you say. In most cities it may be easier to do a man an ill turn than a good one. In this it certainly is so. I think that you yourself are aware of this.'

"We cannot but look with great interest," writes Dr. Whewell, "at this warning menace, when we recollect that this man was the cause of Socrates's death. . . . But we shall also look with interest at Socrates's reply to this menace. He says to his companion :

"'Meno, Anytus seems to be out of humour with me, nor am I surprised at it. For in the first place he thinks that I accuse the eminent men of whom I speak as having done something wrong ; and then he thinks that he himself is one of these eminent men. If he ever come to know what it really is to be ill spoken of, he will not be angry at such expressions as these ; but at present he does not know.' "*

When the philosopher was serious and opened—"I know not," said Alcibiades, "whether any one of you has seen the images within ; but I once saw them, and they appeared to me to be all golden, and all beautiful and wonderful, that I thought I must in short do whatever Socrates ordained." Need we wonder that with such a teacher Plato uses the expression, "*the madness and Bacchic fury of philosophy ?*"

Further, Socrates is described as unequalled for prudence and self-control, deliberately brave in battle, and enduring better than all others the severities of campaigning. So hardened had his frame become by his temperate habits that, while others were starving, he on the same diet remained robust and cheerful ; while others could only resist the cold when wrapped in many clothes, he moved about bare-footed and in his ordinary attire. And when he was in deep thought, we learn that he would remain long fixed in one posture, entirely indifferent to the passage

* Page 243.

of time, or to the things which transpired around him. His discourses are said to have appeared at first very ridiculous, being enveloped in such nouns and verbs that they might be compared to the hide of a Satyr. But he who beheld these discourses opened and got within them, found that they alone of all other discourses possessed an internal meaning; and that they were most divine, and held the most numerous images of virtue extending to the furthest point, or rather to everything which it was fitting for him to consider who intended to become a man at once beautiful and good.*

Let us gather a few fragments from these wonderful discourses as Plato depicts them, and from them learn somewhat more of the discourser and his great pupil. In the *Lysis*, or dialogue on Friendship, Socrates is represented gossiping with lads, and at one part he remarks, "See, Hippothales, how one ought to talk to a boy; taking him down and bringing him to reason, not blowing him up with conceit and spoiling him, as you do." The purport of this dialogue, Dr. Whewell remarks, is to show "that the way to win a boy's regard and respect is to talk to him so as to set his mind to work; and that he will like this better than high-flown praises and literary terms of expression."†

Again, when Socrates has thoroughly perplexed, with a geometrical question, the boy in the dialogue on Virtue, he observes, addressing Meno :—

"Soc. In bringing him to a state of perplexity and *benumbing* him as you call it, like the numbing-fish, have we done him harm?

"Men. It does not appear to me that we have.

"Soc. No. We have done something in the way of preparation, it would seem, to show what is his real position. For, at present he would willingly seek what he does not know: but in his former disposition he would without scruple have asserted to a numerous audience and upon many occasions, (and have thought that he was talking wisely,) that the line must have a double length.

"Men. Very likely.

"Soc. Do you think that he would have set about trying to seek or to learn that which he thought he knew and did not know, before he was brought into this state of perplexity by being aware that he does not know, and so led to desire to know?—Men. I think he would not, Socrates.

"Soc. So he was the better for being benumbed?—Men. It seems so."

Then we turn to an illustration of the philosopher's mode of dealing with adults. In the *First Alcibiades*, the dialogue on the Nature of Man, Socrates says :—

"'To educate ourselves we must improve ourselves. But we must

* The *Banquet*.

distinguish. We may improve a thing, or improve what belongs to a thing. Shoes belong to the feet, the cobbler improves shoes. But Gymnastic improves the feet. So that to improve ourselves, and to improve what belongs to us, are different operations, belonging to different arts.'

"Socrates then goes on to pursue this notion. 'How,' he asks, 'are we to fix our attention on the thing itself as distinguished from what belongs to it? We must distinguish between the person and the instruments that he uses. The leather-cutter and the lyrist use the knife and the lyre, but *they* are something different from *these.* They use also their hands and their eyes, but yet *they* are not *these.* The man is something different from the parts of his body. What then is the man?'

"Socrates then goes on: 'The soul uses the body as an instrument; commands it as a servant. The man must be either the Soul or the Body, or the compound of the two. He is not the Body, for the Body is governed by the Soul. He is not the compound of the two, the part governed and the part governing. It must be the governing part—the Soul. When Socrates converses with Alcibiades, it is their souls which converse. And thus, when the Delphic oracle bids us know ourselves, it bids us know our Souls. When I admire and love Alcibiades, I love his Soul. Those who loved merely the body of Alcibiades did not love him. Those lovers left you when the body lost the bloom of youth; and therefore it is that I alone stick to you when they have all deserted you. And this is the solution of the question which, when we began, you said you were going to ask me.

"'And now my care for you is, that you may not be spoiled by the People of Athens, and become a popularity-hunter;—the ruin of many promising men. And to avoid this, cultivate your soul, and then you may go into public life carrying with you an antidote to every danger.'

"There is then use made of an analogy of a very lively kind, to illustrate what is meant by knowing ourselves. 'We may take,' Socrates says, 'the analogy of the eye. The eye sees not itself but by reflection from some other thing; for instance a mirror. But the eye can see itself also by reflection in another eye; not by looking at any other part of a man, but at the eye only. So too the Soul, to know itself, must look into the Soul of a friend; into the knowing, the wise part of the Soul. There is nothing more divine than this. We shall thus know our faults, and our good faculties; we shall thus acquire Sophrosyne, true wisdom, the virtue of the Soul.'

"'Moreover,' Socrates adds, 'he who does not know himself cannot know others. He cannot direct a city; he cannot even direct a household. He cannot know what it is that he does. He must err. And he who errs, does ill; and he who does ill is unhappy. It is not the rich man who is happy, but the truly wise—the *Sophron.* It is not walls, and docks, and ships, which cities require, in order to be happy, nor numbers, nor greatness, but virtue. If you are to manage well the affairs of the city, you must make the citizens virtuous. And no man can give what he has not. You must *be* virtuous. You must get justice and wisdom. You must act, regarding the divine part of your nature, as we have just called it. Then you and the city will do well and be happy.' "

Of his own knowledge Socrates speaks in this manner in the *Apology*. "I reasoned thus: I *am* wiser than this man; for it is tolerably plain that neither of us knows what is right and good; but he thinks he does know; I, as I do not know, do not think I know. I have this small advantage over him, that what I do not know, I do not think that I know."* This is the true pole-star of philosophical knowledge.

Again: "If I am wise in anything, it is in this, that as I know nothing of the state of departed spirits, so I do not *think* I know; but that to do wrong, and to disobey good guidance, whether of God or man, is an evil and a disgrace, that I know."† So also he tells us that in seeking wisdom he found that of those individuals who had mastered their own art, "each thought that he was also very wise in other things of the greatest moment; and this conceit of theirs spoilt their wisdom. So I asked myself whether I had rather be as I was, not possessing their knowledge and not having their ignorance, or to have both as they had. And I answered to myself and to the oracle, that it was better for me to be as I was."‡

He aimed solely at a life of virtue, and he tells us in the *Apology* that he had shown in deeds, not words, that "he cared not a jot for death, if he might be allowed a rough expression, but he cared mightily about doing nothing unjust or wicked."§ Again, he exclaimed, "The great object, O men, is not to escape death, but to escape baseness and wickedness; Wickedness runs faster than Death, and so is more difficult to escape."|| And again, when the day of execution was nigh at hand, Crito urged him to respect the opinions of the many, to which he was about to be sacrificed, and save his life, but Socrates answered:—

"'Now pray, Crito, was this well said? You, according to all human appearance, are in no danger of dying to-morrow, and therefore the impending calamity need not disturb your judgment. Use your judgment then. Does it not seem to you to be a proper saying that we are not to respect *all* opinions of men, but to respect some and not to respect others; and not to respect the opinions *of* all, but to respect those of some, and not those of others. How say you? Was not this well said?'—Cr. It was well said.

"Soc. That we must respect good opinions, and not respect bad ones?—Cr. Yes.

"Soc. And good opinions are the opinions of the wise; bad opinions those of the unwise?—Cr. Of course.

"Soc. But come; how was this followed out? A man who is practising gymnastic, does he attend to the opinion—the praise or

* Page 302. † Page 313. ‡ Page 303. § Page 317. || Page 327.

blame—of any one, or only of a particular person, the master of gymnastic or the doctor of medicine?—Cr. Of him alone. . . . . . .

"And thus we must not consider what the many will say of us, but that one Judge of right and wrong and Truth herself. And thus, Crito, you were mistaken in referring me to the opinion of the many about these points of right and good and honourable.

"But some one may say, These, the many, have it in their power to put us to death. True, my friend, but still we come back to the same point, to which we have often come before. Do we still hold to our principle that the main point is, not to live, but to live well?—Cr. We hold to that.

"Soc. And to live well is to live rightly and honourably: does that stand?—Cr. That stands."

We are, moreover, instructed in the *Charmides,* the dialogue on Sound-Mindedness, as Dr. Whewell renders the word *Sophrosyne,* "that it was not living according to knowledge which made us live well and happily, not even if you put all other knowledges together; but according to that one knowledge, the knowledge *of good and evil.* If you take away that knowledge, the other knowledges may still remain, but they will be of small use to us. And if *Sophrosyne be* the knowledge of knowledges, the valuable thing is not that, but the knowledge of good and evil."* Further, we learn from Xenophon, that Socrates refused to recognise a distinction between wisdom and virtue; "for, he said, that he who knew what was vile, and avoided it, was wise and virtuous."

And so it was, that when the great-hearted old man was confronted with his judges, death weighed with him but as a feather in the balance, as compared with what he deemed was right, and therefore not a whit does he vary from his accustomed blunt mode of speaking, or for a moment does he conceal his ordinary train of thoughts—aiding his persecutors rather than defending himself, and aggravating rather than soothing the judges and the people.

"'And so, Athenians, I am very far from delivering a defence of myself; I am defending you;—defending you from condemning me because I use the gift which God has given me. For if you put me to death, you will not readily find any one who will fasten himself upon the city (to use a comparison which may seem to you odd, but which is very just), like a rider upon a horse, powerful and of good blood, but heavy and sluggish, and needing to be roused by the spur. I seem to be appointed by the God such a rider to this city, sitting close to you, and exciting you by persuasion and reproach, all day long without ceasing. Such another, I say, you will not readily find; and if you will take my advice, you will not destroy me. Perhaps you may be like persons who are angry because one awakes them when they are

* Page 66.

sleepy, and may shake me off, as Anytus bids you, and kill me; and then you may go on sleeping for the rest of your lives, except God in his care for you send you another like me.' "*

Xenophon tells us that after Socrates had finished his defence, " he went away with a radiant look and a steady step, such as suited the tone which he had taken. And when he perceived that those who accompanied him were weeping, 'What is this?' he said. 'Do you weep now? Did you not know that from the time of my birth nature had condemned me to death? And if I were now going, by death, to lose good things which are flowing in upon me, both I and my well-wishers might weep. But if I part with life when I have only evils to look forward to, I think you ought all to rejoice as if a fortunate thing had happened to me.' One Apollodorus, who was present, a great admirer of his, but in other respects a simple person, said, 'This, O Socrates, is the hardest thing to bear, that I see you put to death wrongfully.' And he, stroking the youth's head, replied, 'My dear Apollodorus, should you have liked better to see me put to death justly?' and smiled."

Then, thirty days after, and when the time of execution was nigh at hand, we see Crito in the early morning sitting by the bed-side of the sleeping philosopher. Presently Socrates awakes, and beholding Crito sitting there, exclaims, " How was it that you did not rouse me, but sat in silence by my side?" To which Crito responds, " God forbid that I should do that! I should be very sorry to be waked when in such sorrowful case. But I have been admiring you, seeing how soundly you sleep. I purposely abstained from waking you, that what time you have before you you may pass as lightly as may be. Often in the previous course of my life I have admired your happy temper, but never so much as now in your present calamity, to see how quietly and cheerfully you bear it." Socrates rejoins, " Why, Crito, it would be very unreasonable, at any age, to be vexed because one must die." And so to the end. The morning of the day on which he must drink the fatal draught dawns. His disciples and friends are around him, sorely oppressed with grief. We see Xantippe sitting beside him, with one of his children in her arms, and we hear her frantic grief as she is led away from the prison. We look with wonderment on the strange equanimity of Socrates, as, on the brink of the grave, he sits quietly on the edge of the bed, and, freed from his bonds, draws up his leg, and rubbing it, playfully remarks, " How strange a thing is that, my friends, which is called pleasure! and how oddly is it connected with its supposed opposite, pain! Pleasure and pain do not come to man together, but if a person runs after the one and catches it, he almost in-

* *Apology*, p. 315.

evitably catches the other too, as if they were fastened together at one end." Then follows that marvellous dialogue,* in which Socrates asserts the proposition that philosophy is nothing else than a preparation of the soul for death, and argues the immortality and nobility of the soul; and we still delightedly listen to, rather than read—so vividly has Plato depicted the scene, and so little do we care to hear how much may depend upon the dramatic power of the writer, how much is a literal record of events—the profound and exquisitely illustrated reasonings. Hearken to a few phrases :—

"'You (Cebes and Simmias) seem as if you would willingly have the proof a little further explained. You seem to be frightened, as children are, that when the soul passes out of the body, the wind may blow it quite away and disperse it entirely, especially if there be strong breezes stirring when the man dies.' At this Cebes laughed, and said: 'Well, Socrates, suppose that we are frightened; and do you encourage and comfort us. Or rather, suppose, not that *we* are frightened, but that there is a child within us who is so. Let us try to persuade *him* not to fear death, as a kind of bugbear or hobgoblin.' 'Yes,' said Socrates: 'and to do this, we must use some charm, that we can sing over him day by day, till the incantation has quite dispelled his fears.' "† . . . .

"'When the soul regards objects by the aid of the senses, and thus *uses the body* in its contemplation of the world, it is disturbed and distracted by contact with the body. It wanders, and grows giddy as if intoxicated. But when it considers objects by the help of its own powers alone, it is then drawn to that which is pure and eternal and immortal and uniform, and feels that it is of the nature of that. Its wanderings end; it becomes steady and uniform like its objects: and this condition is called *Wisdom.*' "‡ . . . .

"'Those who truly pursue philosophy, abstain from the gratification of bodily desires, and bear all trials, and resist all temptations; they fear no privations and no poverty, like common men who are enslaved by the love of wealth. They fear no obloquy nor loss of good name, like those who are carried away by the love of honours and of power. They leave such men to go their way, and heed them not. They care for their souls, not their bodies, and take another course. They reckon that such persons do not know to what they are tending. They will not run counter to philosophy and her teaching;—they aim at the liberation and purification which she gives, and follow where she leads.

"'You ask how they do this? I will tell you. Those who really love truth know how philosophy benefits the soul. They know that she receives it completely bound up in and fastened to the body; compelled to look at everything, not directly, but as it were through the walls of a prison; and thus condemned to darkness, and feeling that the strength of its prison consists in the strength of its own de-

* See *Phædo.* † Page 388. ‡ Page 391.

sires, and that it is itself the accomplice of its own captivity. They know that philosophy receives the soul thus entangled, and comforts it, and sets about liberating it; by showing it that perception by the eyes and by the ears is full of deceit; by persuading it to trust these as little as possible, and to collect itself into itself, and to trust its own peculiar and innate powers of contemplating realities: to ascribe no reality to what it apprehends in any other way: since all such things are the objects only of external sense and vision, but the things which it sees directly and by itself are invisible and intelligible only. The soul of a real lover of truth does not oppose itself to this offer of liberation; and hence abstains from pleasures and desires and griefs and fears with all its power; for it considers that when a man is under the sway of strong joy or fear or grief or desire, the evils which thus move him are not so great as he imagines; while the last and greatest of evils he suffers without regarding it:—namely, the belief that visible things, the objects of these joys and griefs, are the clearest and strongest of realities, and the consequent subjugation of its powers to them. Every pleasure and every grief furnishes a nail which fastens the soul to the body; makes it an appendage to the body, and like the body; judging of things as the body judges.' "*

Let us not overlook that episode when Socrates, laying his hand upon the head of Phædo, who sat on a low seat by the bed-side, and stroking his hair which lay upon the neck, an act he often did, said:—

" 'Phædo, I suppose you intend to cut off these beautiful locks to-morrow, as a sign of mourning.'—'So it seems, Socrates,' I replied.—'Do not do it *then*,' said he, 'if you will take my advice.'—'What do you mean?' said I.—'You must cut your locks and put yourself in mourning *to-day*, and I must do the same, if our Doctrine is mortally stricken and we cannot bring it to life again. If I were you, and if this Doctrine of the Immortality of the Soul were conquered, I would take an oath, as the Argives did, never to let my hair grow, till in a fresh fight I had overcome the arguments of Simmias and Cebes.'—'But,' said I, 'according to the proverb, even Hercules is not a match for two.'—'Well,' said he, 'take me for your Iolaus, the companion of Hercules, while daylight still allows you to do so.'—'I take you for my aid,' said I, 'not as Hercules took Iolaus, but as Iolaus took Hercules.'—'It comes to the same thing,' said he. 'But there is one error which we must take care to avoid.'—'What is that?' said I.—'The error of coming to dislike Reason, as some persons come to dislike men, and become misanthropes. There can be no greater misfortune than to hate Reason. And the hatred of Reason may be got in the same way as some get a hatred of men. Misanthropy is produced by trusting some man entirely, without knowing mankind, and believing him to be true and sound and honest, and then finding him false and dishonest; and then doing the same thing to another, and another. When this has happened to a man often, and especially if it have been among those whom he deemed his surest friends, at last he hates everybody, and thinks that nobody is honest. Have you not observed

* P. 395-6.

this?'—'Certainly,' said I.—'And is it not,' said he, 'a shocking result? And it is plain that it comes of a man dealing with men without a knowledge of mankind. Now arguments are in this respect like men. If a man assent to an argument as true, without knowing how to reason, and then shortly after find it to be false, sometimes when it is so, sometimes when it is not; and so of another and another; you know that he comes to mistrust all argument. Especially those who are most occupied with arguing on both sides of questions, you know that at last they think they are very wise, and can see, what others cannot see, that nothing is solid and certain;—that everything runs upwards and downwards like the currents of the Euripus, and that nothing is permanent and stable.'—'You say very truly,' said I.—'Would it not then,' said he, 'be a lamentable thing, if, when an argument was really solid and intelligible, a person who had been engaged among inconclusive reasonings, which leave no stable conviction, and had thus become sceptical about the sound argument, should blame, not himself and his own bad reasonings, but Reason itself; and should take to speaking ill of it, and thus lose the benefit of truth and knowledge.'—'A lamentable thing indeed,' said I.—'First then,' said he, 'let us take care to avoid this error; and not admit the belief into our minds that there is nothing sound and certain in itself. Let us rather suppose that our minds are not sound, and let us try manfully to make them so:—you and the rest, because you have long to live, and I, because I am soon to die: that I may behave as becomes a philosopher, and not like mere disputatious talkers. They in their disputes do not care on which side the truth lies, but merely try to persuade the bystanders to adopt the opinions which they have asserted. I am in a very different state from them. My main purpose is, not that I may convince the bystanders, except as a secondary object, but that I may satisfy myself. And see, my dear friend, under what advantages I am reasoning. If my doctrine is true, it is well to know it; and even if after death there be nothing, I shall still avoid wearying my companions with my lamentations while I live. And my error will not last long: there will soon be an end of it. And with this preparation, O Simmias and Cebes, I come to the argument. And you, if you will take my advice, will think little about Socrates, but a great deal about Truth; and if I say what seems to be true, take it up; but if otherwise, reject it; being on your guard that I may not, in my eagerness, deceive you as well as myself, and thus depart like a bee, leaving my sting in you.'"*

Thus conversing, the end draws nigher and nigher, but there is no wavering in the calm confidence of Socrates in the final happiness of the virtuous man, and the belief that when death comes to man his mortal part dies, but the immortal lives, and thus our souls shall exist in another world. "It is right," he also urges, "to bear this in mind: that if the soul be immortal, it requires our care, not only during the time that we call life, but for all time; and great is our danger if we neglect it. If death were the end of all, it would be a gain for the wicked to get rid of

* P. 405-8.

their body and of their wickedness at the same time, when their soul departs. But since the soul is immortal, there is no help for it except to make it good and wise: for it carries nothing with it into the other world, but the preparation which it has received here."*

The fatal moment has come; Socrates, saying gentle and kindly words to those who are weeping around him, and to his executioner who adds his tears to the rest, takes the cup, drinks the deadly potion, and dies, "of all the men we have known," says Plato, "the best, the wisest, and most just."

In depicting Socrates we have adhered chiefly to the literal text of the Platonic Dialogues, as rendered in Dr. Whewell's version, thinking that in this manner we should best convey to our readers a notion of the merit of that version, and an inkling (if such be needed) of the charm which Plato's writings possess, as well as of his representation of the Socratic method of thought.

---

